# Improvement in ICU Mortality From Sepsis Associated With Recuperation From Septic Multiple-Organ Failure: A Retrospective, Single-Center, Cohort Study

**DOI:** 10.7759/cureus.57118

**Published:** 2024-03-28

**Authors:** Kenichiro Kikuchi, Satoshi Kazuma, Yoshiki Masuda

**Affiliations:** 1 Department of Anesthesiology, Sapporo Medical University School of Medicine, Sapporo, JPN; 2 Department of Intensive Care Medicine, Sapporo Medical University School of Medicine, Sapporo, JPN

**Keywords:** multiple organ failure, intensive care unit, mortality, immunosuppression, septic shock, sepsis

## Abstract

Background: Although mortality due to sepsis has decreased in recent decades, there are few studies on the timing of death during ICU stay. Characteristics of patients related to changes over the years of ICU death and changes in the timing of ICU death will provide new insights for future sepsis management.

Methods: This was a single-center, retrospective study. Patients admitted to the ICU for sepsis between 2005 and 2019 were included in the study. The study period was divided into three five-year intervals, and the timing of death in the ICU was divided into early-stage (1-3 ICU days), mid-stage (4-14 ICU days), and late-stage (15 or more ICU days). Patient characteristics related to ICU death at three five-year intervals and the timing of death were evaluated.

Results: ICU mortality for sepsis has decreased over time (2005-2009, 30.2%; 2010-2014, 21.0%; 2015-2019, 12.1%; p<0.01). In the timing of death, only mid-stage mortality decreased. Multiple-organ failure (OR, 4.53; 95% CI, 2.79-7.48) and hematological malignancies (OR, 2.48; 95% CI, 1.19-5.07) were associated with ICU mortality over entire study periods. Only multiple-organ failure was associated with ICU mortality at the five-year intervals (OR, 5.94; 95% CI, 2.73-13.7 for 2005-2009; OR, 4.01; 95% CI, 1.82-9.31 for 2010-2014; OR, 2.58; 95% CI, 1.05-6.59 for 2015-2019). Mid-stage mortality of multiple-organ failure decreased (2005-2009, 12.8%; 2010-2014, 7.6%; 2015-2019, 1.6%; p=0.02). However, early- and late-stage mortality of multiple-organ failure did not change.

Conclusions: Improvement in mid-stage mortality in septic patients with multiple-organ failure can contribute to the improvement of overall ICU mortality in patients with sepsis.

## Introduction

The widespread use of standardized treatment strategies for sepsis has led to a decrease in mortality due to sepsis and septic shock in recent decades [1]. Adherence to guideline-based sepsis bundles, early recognition of sepsis, and enlightening medical staff regarding sepsis could have improved the prognosis of sepsis [2-5].

Guidelines for the management of sepsis and septic shock help professionals and non-professionals make appropriate decisions regarding the diagnosis and treatment of sepsis and septic shock. Adherence to guidelines was lower in hospital-onset sepsis than in community-onset sepsis [6]. The Rapid Response System (RRS) helps healthcare professionals identify early deterioration in the condition of patients. RRS leads to early recognition and therapeutic intervention for sepsis in hospitalized patients and improves adherence to the sepsis bundle. Achieving the overall sepsis bundles, obtaining blood cultures, and measuring blood lactate reduced mortality [7]. Delays in antimicrobial administration, multiple-organ failure (MOF), cancer, persistent hypotension, and hyperlactacidaemia were associated with a poor prognosis of sepsis [6,8,9]. A dysregulated host response to infection causes the onset and progression of organ dysfunction. Progression of organ damage was associated with increased mortality [10-12]. Control of the source of infection, such as antibiotic therapy or drainage, along with the management of individual organs that maintain oxygen supply and demand, reduces exacerbation of organ damage.

The risk factors for mortality in sepsis are well known [6,8,9]. However, little is known about the association between patient characteristics and timing of death. Evaluating changes in the timing of death in the ICU and the characteristics of patients with sepsis associated with death in the ICU may provide new insights into future management strategies. The aim of this study was to identify important factors for predicting the prognosis of patients with sepsis and septic shock based on changes in the timing of death from sepsis.

## Materials and methods

Patients and methods

We conducted a retrospective study using data from the medical records of the ICU. All patients admitted to the ICU from the wards and diagnosed with sepsis and septic shock (defined according to the Sepsis-3 criteria) [13] from January 2005 to December 2019 were included in this study. In contrast, patients readmitted to the ICU within 72 hours of discharge and those under 15 years of age were excluded. 

Data from ICU admission were evaluated for patient characteristics (age, sex, comorbidities, infectious focus, causative microorganism, positive blood culture, organ failure, and MOF) and the following severity scores: Acute Physiology and Chronic Health Evaluation (APACHE) II score and Sequential Organ Failure Assessment (SOFA) score. Organ failure was defined as a SOFA score of 3 or more for each organ, and MOF was defined as the presence of two or more organs with a SOFA score of 3 or more [12]. Treatments for sepsis (antibiotics within three hours of suspected sepsis, mechanical ventilation, steroids, vasoactive agents, continuous renal replacement therapy, endotoxin absorption therapy, and anticoagulants) after ICU admission were also evaluated. The study period was divided into three five-year intervals: 2005-2009, 2010-2014, and 2015-2019. For each five-year period, we evaluated the early-stage (1-3 ICU days), mid-stage (4-14 ICU days), and late-stage (15 or more ICU days) mortality rates. The ICU, 28-day, and hospital mortality rates for each period were also calculated. 

The characteristics of patients associated with death in the ICU were investigated for the overall study period, and the obtained characteristics were examined for their association with death at three five-year intervals. We also investigated the changes in the obtained characteristics by the time of death (early-stage, mid-stage, and late-stage) over a five-year period. This study was approved by the Institutional Review Board of Sapporo Medical University, Sapporo, Japan (approval number: 332-30).

Statistical analysis

Data are presented as percentages and numbers, means with standard deviations (SDs) or proportions, and 95% confidence intervals (CIs). Chi-squared tests, Fisher's exact tests, one-way ANOVA, and Kruskal-Wallis tests were used to test differences. We performed multivariate analyses using a logistic regression model to investigate the characteristics of patients in predicting death during the overall study period. The obtained factors were used as independent variables to examine their association with death at three five-year intervals. Odds ratios (OR) were reported with 95% CIs. Statistical significance was set at p<0.05. Statistical analyses were performed using the GraphPad Prism 10 software (GraphPad Software, La Jolla, CA, USA). 

## Results

A total of 496 patients were enrolled in this study. Three patients were excluded from this study. There were no significant differences in age, sex, comorbidities, APACHE II score, and the number of patients with MOF among the three periods. The SOFA score at ICU admission was lower in 2015-2019 than in 2005-2009 (p=0.014). Respiratory, abdominal, urinary tract, and catheter-related bloodstream infections (CRBSI) differed among the three periods. Gram-negative infections were significantly more common cause in 2015-2019 than in 2005-2009 and 2010-2014. There was a significant difference in any individual single-organ damage among the three periods. However, no significant difference was observed in the percentage of patients with MOF. The percentage of patients who received antibiotics within three hours of sepsis diagnosis increased, and those who required mechanical ventilation decreased. The ICU, 28-day, and hospital mortality rates decreased during the three five-year periods (Table 1).

**Table 1:** Patient characteristics APACHE: Acute Physiology and Chronic Health Evaluation; SOFA: Sequential Organ Failure Assessment; CRBSI: catheter-related bloodstream infection; GNR: Gram-negative rods; GPC: Gram-positive cocci; CRRT: continuous renal replacement therapy; MOF: multiple-organ failure

Characteristics		Overall	2005-2009	2010-2014	2015-2019	P-value
Sepsis, n (%)		496 (15.5)	149 (14.8)	157 (14.9)	190 (16.7)	0.39
Age, mean (SD)		65.4 (15.1)	65.6 (11.6)	66.2 (14.1)	67.2 (9.2)	0.68
Sex (male/female)		319/177	97/52	109/48	113/77	0.15
APACHE II score, mean (SD)		21.2 (6.2)	20.4 (6.4)	21.6 (5.7)	21.7 (6.4)	0.34
SOFA score, mean (SD)		8.3 (3.3)	8.5 (3.3)	8.4 (3.1)	7.8 (3.2)	0.015
Comorbidities						
Congestive heart failure		22	6	7	9	0.96
Severe diabetes mellitus		36	4	12	20	0.016
Neurological disorder		12	4	5	3	0.66
Chronic kidney disease		33	5	15	13	0.089
Solid or metastatic tumor		133 (26.8)	34 (22.8)	49 (31.2)	50 (26.3)	0.25
Hematological malignancy		47	12	17	18	0.7
Immunosuppression		102 (23.4)	30 (20.1)	39 (24.8)	47 (24.7)	0.53
Liver disease (cirrhosis)		20	8	4	8	0.46
Infectious focus						
Respiratory		160	59	62	39	<0.0001
Abdomen		169	37	55	77	0.0091
Urinary tract		36	5	8	23	0.0053
Skin and soft tissue		64	12	21	31	0.069
CRBSI		17	5	1	11	0.02
Central nervous system		8	2	5	1	0.2
Miscellaneous		15	7	4	4	0.4
Causative microorganisms						
GNR		149	23	35	91	<0.0001
GPC		153	44	41	68	0.45
Fungus		28	8	8	12	>0.99
Miscellaneous		207	78	80	49	<0.0001
Blood culture positive, n (%)		161 (32.5)	36 (24.1)	40 (25.5)	85 (44.7)	<0.0001
Organ failure						
Acute respiratory failure		236	83	91	62	<0.0001
Coagulopathy		190	32	76	82	<0.0001
Acute kidney injury		227	47	91	89	<0.0001
Encephalopathy		73	12	39	22	<0.0001
Liver dysfunction		75	22	34	19	0.011
Shock, n (%)		291 (58.7)	65 (43.6)	88 (56.1)	138 (72.6)	<0.0001
Mesenteric ischemia		12 (2.4)	4 (2.7)	4 (2.5)	4 (2.1)	0.94
MOF, n (%)		203 (40.9)	66 (44.3)	64 (40.8)	73 (38.4)	0.55
Treatment						
Antibiotics within three hours, n (%)		471 (95.0)	130 (87.2)	153 (97.5)	188 (98.9)	<0.0001
Mechanical ventilation, n (%)		354 (71.4)	119 (79.9)	113 (72.0)	122 (64.2)	0.0065
Steroids, n (%)		178 (35.9)	55 (36.9)	49 (31.2)	74 (38.9)	0.32
Vasoactive agents, n (%)		351 (70.8)	98 (65.8)	105 (66.9)	148 (77.9)	0.02
CRRT, n (%)		228 (46.0)	54 (36.2)	87 (55.4)	87 (45.8)	0.0035
Endotoxin adsorption, n (%)		199 (40.1)	60 (40.3)	75 (47.8)	64 (33.7)	0.029
Anticoagulant, n (%)		199 (40.1)	55 (36.9)	63 (40.1)	81 (42.6)	0.56
Mortality						
ICU, n (%)		103 (20.8)	45 (30.2)	35 (22.3)	23 (12.1)	0.0002
28-day, n (%)		138 (27.8)	55 (35.7)	49 (31.2)	34 (17.9)	0.0002
Hospital, n(%)		195 (39.3)	68 (45.6)	65 (41.4)	62 (32.6)	0.042

In multivariate logistic regression analyses, MOF at ICU admission and hematological malignancy were associated with ICU mortality (OR, 4.53; 95% CI, 2.79-7.48; OR, 2.48; 95% CI, 1.19-5.07, respectively) for the overall study period. In the three five-year intervals, only MOF was associated with ICU mortality (OR, 5.94; 95% CI, 2.73-13.7 for 2005-2009; OR, 4.01; 95% CI, 1.82-9.31 for 2010-2014; OR, 2.58; 95% CI, 1.05-6.59 for 2015-2019) (Table 2).

**Table 2:** Multivariate analysis of predisposing factors for predicting ICU mortality in the overall study period and three five-year intervals MOF: multiple-organ failure

Independent variables	Overall (n=496)		2005-2009 (n=149)		2010-2014 (n=157)		2015-2019 (n=190)	
	Survive/death (mortality%)	Odds ratio (95% CI)	Survive/death (mortality%)	Odds ratio (95% CI)	Survive/death (mortality%)	Odds ratio (95% CI)	Survive/death (mortality%)	Odds ratio (95% CI)
Age, mean (SD)	65.3/65.5 (15.6)	1.005 (0.99-1.022)	-	-	-	-	-	-
Male sex, n (%)	250/63 (63.6)	1.12 (0.68-1.86)	-	-	-	-	-	-
MOF at ICU admission, n (%)	130/73 (36.0)	4.53 (2.79-7.48)	33/33 (50.0)	5.94 (2.73-13.71)	38/26 (40.6)	4.01 (1.82-9.31)	59/14 (19.2)	2.58 (1.05-6.59)
Shock, n (%)	217/60 (55.2)	1.17 (0.72-1.91)	-	-	-	-	-	-
Immunosuppressive, n (%)	71/37 (18.1)	1.73 (0.86-3.39)	-	-	-	-	-	-
Solid tumor, n (%)	113/20 (28.8)	1.07 (0.61-1.84)	-	-	-	-	-	-
Hematological malignancy, n (%)	29/18 (38.3)	2.48 (1.19-5.07)	5/7 (58.3)	2.47 (0.68-9.64)	11/6 (35.3)	1.71 (0.52-5.16)	13/5 (27.8)	2.89 (0.82-8.83)
Mesenteric ischemia, n (%)	17/12 (4.3)	2.10 (0.79-5.25)	-	-	-	-	-	-
Neurological disorder	15/4 (3.8)	1.66 (0.50-7.14)	-	-	-	-	-	-

The mid-stage ICU mortality significantly decreased over the five-year intervals (p<0.001). Early- and late-stage deaths did not change over the three five-year intervals (Table 3).

**Table 3:** Changes in the timing of ICU death over three semesters

Timing of ICU death	Overall (n=469)	2005-2009 (n=149)	2010-2014 (n=157)	2015-2019 (n=190)	P-value
Death in the ICU, n (%)	103 (22.0)	45 (30.2)	35 (21.0)	23 (12.1)	<0.001
Early-stage (≦3 days), n (%)	33 (6.7)	11 (7.4)	10 (6.4)	12 (6.3)	0.69
Mid-stage (4-14 days), n (%)	48 (9.7)	26 (17.4)	16 (10.2)	6 (3.2)	<0.001
Late-stage (>15 days), n (%)	22 (4.4)	8 (5.4)	9 (5.7)	5 (2.6)	0.3

**Figure 1:**
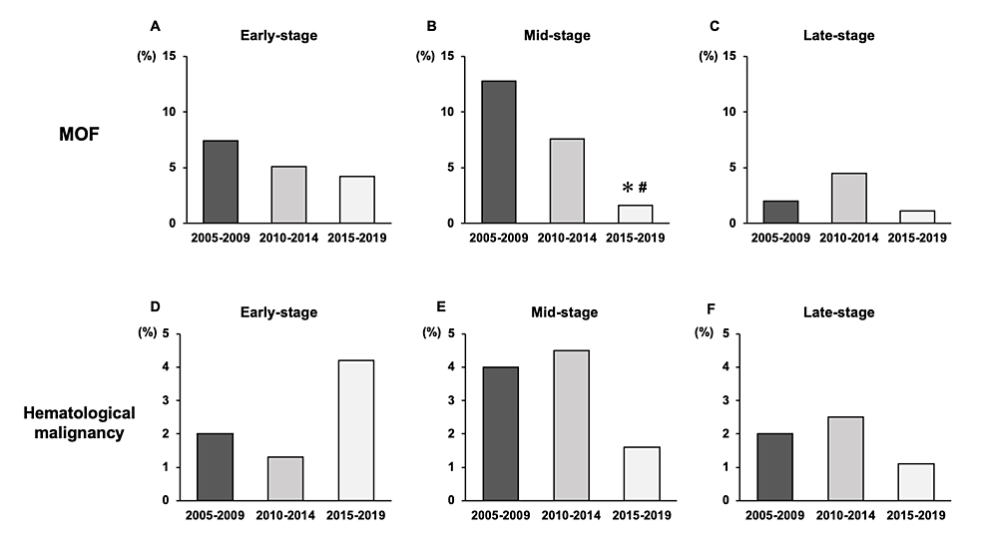
Changes in the timing of death in patients with MOF and hematological malignancies (A-C) Percentage of early-, mid-, and late-stage deaths in patients with MOF. At the mid-stage, mortality in patients with MOF was lower in 2015-2019 than in 2005-2009 and 2010-2014. (D-F) Percentage of early-, mid-, and late-stage deaths of patients with hematological malignancy ✻: p<0.0001 vs 2005-2009; #: p=0.007 vs 2010-2014; MOF: multiple-organ failure

The mid-stage death rate of patients with MOF significantly decreased (2005-2009 vs 2015-2019, p<0.0001; 2010-2014 vs 2015-2019, p=0.007), and that of hematological malignancy did not change over the three five-year intervals (Figure 1).

## Discussion

Mortality due to sepsis decreased in all three five-year periods, from 2005 to 2019. The factors predicting ICU death were MOF at ICU admission and hematological malignancy during the entire study period. Of the two factors obtained, MOF was the only factor predicting ICU death at the three five-year intervals. We also found that only mid-stage death (4-14 ICU days) decreased over the three five-year intervals. Mid-stage death in patients with MOF at ICU admission decreased significantly over five-year intervals.

The ICU, 28-day, and in-hospital mortality rates of septic patients at our institution improved over three five-year intervals. Previous studies have reported that the mortality of septic patients was improved by early effective antibiotic administration or vasopressor initiation [9,14,15]. In the present study, the administration of antibiotics within three hours and the use of vasoactive agents were increased during the same intervals. This finding suggests that resuscitation therapies may have contributed to the decrease in mortality. These therapies may have also contributed to the decrease in the need for mechanical ventilation.

The characteristics of patients who died during ICU stay were MOF and hematological malignancy at ICU admission. The prognosis of patients with MOF is worse than that of patients with single-organ failure. Furthermore, it has been reported that the more organ failure a patient has, the worse the prognosis [10,16-18]. Septic patients with underlying immunosuppressive conditions, such as hematological malignancies, have a poor prognosis [19-21]. Patients with sepsis with these pathological features should be treated with caution.

As for the timing of death, only mid-stage death decreased over the five-year periods (Table 3). Mid-stage death decreased in patients with MOF, but not in patients with hematological malignancies (Figure 1). Law et al. reported that early-, mid-, and late-stage death in patients with septic shock decreased over 20 years from 1994 to 2014 [22]. This result differs from that of the present study. This may be because our study had a different study period, from 2005 to 2019, and showed a lower mortality rate than the previous study. The results of the present study suggest that the decrease in mid-stage deaths due to MOF may have contributed to the decrease in overall mid-stage deaths in the ICU. MOF at ICU admission may be the key to improving the prognosis of sepsis in the ICU. MOF is an important cause of death in septic shock regardless of the timing of death [20]. Early recognition of sepsis to prevent its progression to MOF and appropriate management through a standardized system are important. The establishment of an RRS or prevention of infection for early recognition and adherence to guidelines or a sepsis treatment system for standardized therapy has the potential to prevent the progression to MOF.

There was no change in the early- or late-stage mortality during the study period. The reason for this disparity may lie in the pathophysiology of early- and late-stage sepsis. It is possible that patients who died during early-stage sepsis suffered from a prominent inflammatory response that led to rapid MOF, which can rapidly lead to death [23]. RRSs have been shown to provide an accurate assessment of a patient's condition to allow for appropriate treatment [24], which may contribute to the early recognition of sepsis and improve the prognosis in such patients. The lack of change in late-stage death, coupled with a significant decrease in ICU mortality over the study period, suggests that mid-stage death may have shifted to late-stage death due to the so-called persistent inflammation, immunosuppression, and catabolism syndrome [25], a recent phenomenon that worsens long-term prognosis. Diagnostic methods and therapeutic interventions for persistent inflammation, immunosuppression, and catabolism syndrome have not yet been standardized. Therefore, further studies are needed to elucidate the pathophysiology of late-stage death and discover novel treatment strategies to prevent it.

This study has limitations. This retrospective study covered the period from 2005 to 2019. Because this was a single-center retrospective study, the results of this study cannot be applied to all patients with sepsis. In contrast, the population of patients with sepsis at our institution was not significantly different from those in previous studies [1,22]. The results of this study did not consider therapeutic interventions in the ward prior to ICU admission or interventions by the RRS. The RRS was implemented at our hospital during the study period. The percentage of septic patients with MOF at ICU admission decreased, although not significantly (44% in 2005-2009; 40.8% in 2010-2014; 38.4% in 2015-2019). These factors may have affected the results. 

## Conclusions

ICU mortality due to sepsis decreased at five-year intervals between 2005 and 2019. MOF and hematological malignancy were associated with ICU death during the study period. Only MOF was associated with ICU death at the five-year intervals.

The reduction in mid-stage (4-14 ICU days) mortality for sepsis contributed to the improvement in the overall ICU sepsis mortality. It was suggested that a decrease in mid-stage mortality in patients with MOF at ICU admission was the main reason for the reduction in mid-stage mortality.
